# Investigation of Motor Cortical Plasticity and Corticospinal Tract Diffusion Tensor Imaging in Patients with Parkinsons Disease and Essential Tremor

**DOI:** 10.1371/journal.pone.0162265

**Published:** 2016-09-07

**Authors:** Ming-Kuei Lu, Chun-Ming Chen, Jeng-Ren Duann, Ulf Ziemann, Jui-Cheng Chen, Shang-Ming Chiou, Chon-Haw Tsai

**Affiliations:** 1 Neuroscience Laboratory, Department of Neurology, China Medical University Hospital, Taichung, Taiwan; 2 School of Medicine, Medical College, China Medical University, Taichung, Taiwan; 3 Graduate Institute of Neural and Cognitive Sciences, Medical College, China Medical University, Taichung, Taiwan; 4 Department of Radiology, China Medical University Hospital, Taichung, Taiwan; 5 Institute of Cognitive Neuroscience, National Central University, Zhongli, Taiwan; 6 Institute for Neural Computation, University of California San Diego, La Jolla, California, United States of America; 7 Department of Neurology & Stroke, and Hertie Institute for Clinical Brain Research, Eberhard-Karls-University, Tübingen, Germany; 8 Department of Neurosurgery, China Medical University Hospital, Taichung, Taiwan; University Medical Center Goettingen, GERMANY

## Abstract

Parkinson’s disease (PD) and essential tremor (ET) are characterized with motor dysfunctions. Motor circuit dysfunctions can be complementarily investigated by paired associative stimulation (PAS)-induced long-term potentiation (LTP)-like plasticity and diffusion tensor imaging (DTI) of the corticospinal tract (CST). Three groups of twelve subjects with moderate severity PD, ET with intention tremor and healthy controls (HC) were studied. The primary motor cortex (M1) excitability, measured by motor evoked potential (MEP) amplitude and by short-interval and long-interval intracortical inhibition (SICI and LICI) was compared between the three groups before and after PAS. The DTI measures of fractional anisotropy (FA) and mean diffusivity (MD) were acquired. PAS effects and DTI data were simultaneously examined between groups. PAS increased MEP amplitude in HC but not in PD and ET. SICI and LICI were significantly reduced after PAS irrespective of groups. No significant differences of the mean FA and MD were found between groups. There was no significant correlation between the PAS effects and the DTI measures. Findings suggest that both PD and ET with intention tremor have impairment of the associative LTP-like corticospinal excitability change in M1. The microstructure of the CST is not relevant to the deficiency of M1 associative plasticity in PD and ET.

## Introduction

Parkinson’s disease (PD) and essential tremor (ET) are the most common movement disorders. It is now a widely accepted concept that motor circuit dysfunction underlies motor symptoms in PD and ET. Despite of a distinct pathological basis between these two disorders, some patients may present similar clinical manifestations or superimpose both diseases [1]. Evidence has been provided that the primary motor cortex (M1) is involved in pathogenesis of tremor in PD and ET [2–5]. Previous studies also revealed that PD patients have significant dysfunction of M1 heterosynaptic plasticity which can be non-invasively investigated by paired associative stimulation (PAS) [6]. However, it remains unclear to what extent M1 plasticity is similarly impaired in PD and ET.

Among the complex motor circuits, M1 and its major efferent pathway, the corticospinal tract (CST), play a key role on defining the final motor output. Dopamine depletion in PD not only alters regional metabolism and interneuron activity in M1, but also leads to functional reorganization of motor maps [7]. An animal study revealed evidences that dysfunction of movement-related activity in the lamina 5b pyramidal-tract type neurons which form the primary efferent motor pathway to the spinal cord may be a central factor in the pathophysiology of parkinsonian motor signs [8]. Transcranial magnetic stimulation (TMS) studies also suggested corticospinal hyperexcitability in PD at rest [9]. Neuroimaging studies have demonstrated widespread white matter involvement in early stage idiopathic PD and ET [10, 11]. Nevertheless, it remains unclear how the CST *per se* is affected in PD and ET. Currently it becomes feasible to assess the function and the microstructure of the CST and their relationship by adopting a combined electrophysiological and neuroimaging approach. Although a limited number of studies revealed that CST microstructure may be not significantly impaired in PD and ET by group comparisons [12, 13], the correlation between the microstructure of the CST and the motor cortical excitability on the individual basis remains inconsistent in healthy subjects and largely unknown in patients with PD or ET [14, 15]. Therefore it can be advantageous to simultaneously study the microstructure of the CST and M1 plasticity to clarify the relationship in between.

In this study we investigate PAS-induced long-term potentiation (LTP)-like M1 plasticity and diffusion tensor imaging (DTI) of the CST, which could serve as complementary tools to further elucidate the pathophysiology of these two common neurodegenerative disorders. There are different repetitive TMS protocols capable of inducing LTP-like M1 plasticity. The PAS protocol was chosen because the change of the spike-timing dependent heterosynaptic plasticity induced by PAS can be more sensitive for PD patients than the other protocols [16]. We suppose that findings of the current study may provide further insights to what extent PD and ET have similar or different pathophysiology in M1 and whether the microstructure of the CST is related to the M1 pathophysiology. A part of the findings have been reported as a brief abstract form in the congress book [17] (S1 Text). Here we report the complete findings.

## Methods

### Subjects

Three age-matched groups were recruited in this study. Each group included 12 subjects. The PD group (age, 66.1 ± 8.8 years; 10 male) fulfilled the UK Brain Bank diagnostic criteria (**Table 1**). None of the PD patients had levodopa-induced dyskinesia. The ET group (age, 65.2 ± 8.9 years; 6 male) fulfilled the clinical diagnosis of “classical ET” [18] and showed mild to moderate intention tremor [19] (**Table 1**). All patients were requested to discontinue medications for at least 24 hours prior to the PAS experiment. Twelve healthy controls (HC) without a history of neurological disorders were recruited as the control group (age, 68.9 ± 8.8 years; 6 male). There was no abnormal sign of sensory system in all subjects. Concerning the statistical power, twelve subjects in each group can give a power of 0.95 by assuming a medium effect size of 0.35 [20], three groups (PD, ET, HC) and two measures (before and after PAS). All subjects were right-handed according to the Edinburgh Handedness Inventory. All gave their written informed consent prior to participating in this study, which was conducted in accordance with the latest revision of the Declaration of Helsinki. Approval by the local ethics committee of the China Medical University Hospital was obtained (DMR98-IRB-290).

**Table 1:** Demographic and clinical characteristics of the PD and ET patients.

**PD group (No.)**	**Age (years)**	**Sex**	**Disease duration (years)**	Motor UPDRS /Tremor score*	**Hoehn & Yahr stage**	**More-affected side**	**Medication (daily dose in mg)**
1	67	M	9	18/4	2	R	Levodopa 300, amantadine 300, trihexyphenidyl 3, entacapone 600
2	61	M	3	35/6	2.5	R	Levodopa 200, amantadine 200
3	49	F	2	28/4	2.5	R	Levodopa 600, pramipexole 0.75
4	81	M	2	19/5	2.5	L	Levodopa 200, amantadine 100, trihexyphenidyl 4
5	72	M	5	38/4	2.5	E	Levodopa 600, amantadine 300
6	60	F	0.5	18/8	2	L	Levodopa 200, biperiden 4
7	74	M	2	17/2	2	L	Levodopa 200
8	74	M	1	20/5	2	L	Levodopa 100, trihexyphenidyl 4
9	68	M	2	13/4	2	L	Levodopa 300, ropinirole 0.75
10	69	M	2	19/3	2	E	Levodopa 300, amantadine 100
11	59	M	6	27/17	2	R	Levodopa 300, amantadine 300, pramipexole 0.75
12	59	M	5	27/4	2.5	L	Levodopa 600, amantadine 150, pramipexole 0.75
**ET group (No.)**	**Age (years)**	**Sex**	**Disease duration (years)**	Intention tremor severity (0–3)**		**More-affected side**	**Medication (daily dose in mg)**
1	52	F	> 10	1		E	None
2	62	M	7	1		L	Propranolol 20
3	69	M	> 20	2		E	Clonazepam 0.25
4	50	M	> 10	1		E	Propranolol 30, fludiazepam 0.75
5	63	M	> 10	1		E	None
6	60	M	> 30	2		E	Propranolol 15, clonazepam 0.75
7	81	F	> 20	2		E	None
8	65	F	5	1		E	None
9	65	F	> 20	1		R	Propranolol 20
10	67	F	> 20	2		E	Clonazepam 1
11	74	F	> 20	2		E	Propranolol 30, clonazepam 0.75
12	74	M	> 20	2		E	None

* The score includes assessments for resting and action tremor.

** The score was based on the finger-nose-finger test: 0 = no intention tremor; 1 = probable intention component; 2 = definite intention component; 3 = functionally incapacitated due to intention tremor (Deuschl et al., 2000).

Abbreviations: E: equally affected, F: female, L: left, M: male, R: right

### Procedures

#### PAS

PAS was applied according to a previously established protocol [21, 22]. Comparing to the original PAS protocol which takes 30 minutes [23], this protocol takes only fifteen minutes so it is more tolerable for the patients. It consisted of 225 pairs of electrical stimulation of the right median nerve at the wrist followed by a single TMS pulse over the optimal position (‘hot spot’) of the right-hand abductor pollicis brevis (APB) motor representation of the left M1. The interstimulus interval (ISI) between median nerve and M1 stimulation equaled the individual N20-latency of the median nerve somatosensory-evoked potential (SEP) plus 2 ms. Based on the principle of spike timing-dependent plasticity (STDP), the temporal order of arrival of the median nerve stimulation input and the TMS pulse on M1 results in a significant long-term increase of motor evoked potentials (MEPs) [24, 25]. The ISI was individually adjusted to precisely fit the critical time window of STDP [25]. The individual N20-latency and SEP amplitude N20-P25 were recorded and measured by the method we adopted in the previous study [26]. The electrical stimulus intensity was adjusted to 110% of twitching threshold in the thenar muscle. The frequency of the PAS pairs was 0.25 Hz. The intensity of TMS used for PAS was adjusted to produce MEPs of on average 1 mV in peak-to-peak amplitude in the resting APB when given without conditioning median nerve stimulation. Electrical stimulation was applied through a bipolar electrode (cathode proximal), using constant current square wave pulses (1 ms in duration) at an intensity of three times the perceptual threshold (Digitimer stimulator model DS7A, Digitimer Ltd, England). This PAS protocol induces an LTP-like increase of motor evoked potential amplitude in the APB [21]. TMS was delivered through a focal figure-of-eight stimulating coil (diameter of each wing, 70 mm) connected to two Magstim 200 magnetic stimulators via a Magstim BiStim module (Magstim Co., Carmarthenshire, Wales, UK). The coil was held tangential to the hot spot with the handle pointing backwards and ~45° away from the midline. Resting motor threshold (RMT) and active motor threshold (AMT) were measured at the beginning of the experiment. RMT was defined as the minimum stimulator intensity required to elicit MEPs of > 50 μV in peak-to-peak amplitude in at least 5 out of 10 consecutive trials while the subjects voluntarily relaxed the right APB. AMT was defined as the minimum stimulator intensity required to elicit MEPs of at least 200 μV in 5 out of 10 consecutive trials while the subject maintained a weak voluntary contraction of the right APB (~20% of maximum voluntary contraction). Twenty trials of MEPs of on average 1 mV in peak-to-peak amplitude in the resting APB were obtained before PAS. The same intensity was used for the measurements after PAS. The inter-trial interval varied randomly from 7.5 to 12.5 s to reduce anticipation of the next trial. All subjects were requested to be completely relaxing during the MEP recording. Any trial contaminated by continuous muscle contraction signals (> 500 μV in amplitude) was excluded. For the measurement of short interval intracortical inhibition (SICI), paired pulse magnetic stimuli were applied over the hot spot of the right APB representation in the left M1 [27]. The intensity of the conditioning stimulus was adjusted to achieve ~50% reduction of the test MEP which was set to 1 mV in peak-to-peak amplitude. Twelve trials of paired and single TMS stimuli were recorded in randomized order. The interstimulus interval (ISI) was 2.0 ms [28]. The intensity of the conditioning stimulus was kept the same before and after PAS while that of the test stimulus was adjusted, if necessary, to maintain test MEP amplitudes of on average 1 mV. The range of the adjusted intensity for the test stimulus was less than 5% of the maximum stimulator output. For the measurement of long interval intracortical inhibition (LICI), the settings were similar to those of SICI except the ISI of 100 ms. SICI and LICI were then calculated for each subject and time point by the ratio of the mean conditioned MEP divided by the mean test MEP. The duration of the PAS experiment, including 15 minutes for the PAS intervention, was 1.5 hours.

#### DTI

After the PAS experiment, DTI of all participants was acquired by using a 3.0T GE MR scanner (Signa Excite HDx, Milwaukee, WI, USA) in the other building on the same day. The interval between the two experiments was around 1.5 hours. Given the tradeoff between tolerable scan time for the elder subjects/patients and manageable data quality for fiber tracing, diffusion-weighted, single-shot, spin-echo EPI pulse sequence was used for collecting diffusion tensor images. The images were scanned in transverse section and the scanning parameters were as follows: TR = 15000 ms, TE = minimum (~1.4 ms), matrix = 64 x 64, slice thickness = 4.4 mm, FOV = 240 mm x 240 mm, slice number = ~35 slices, NEX = 2, b = 1000, with parallel speedup factor (ASSET) = 2. The slice number was set differently to properly cover the individual subject’s brain. Twenty-five non-collinear directions with a b-value of 1000 s/mm^2^ and one extra image volume with b-value of 0 s/mm^2^ were used. The DTI data were analyzed using MedINRIA software (http://med.inria.fr/asclepios/software/MedINRIA). In brief, diffusion tensors were calculated for all voxels in the DTI data by computing the three eigenvalues and corresponding eigenvectors. The resulting fractional anisotropy (FA) and mean diffusivity (MD) maps were then computed according to the eigenvalues and further saved in the analyze format for computing the mean FA and MD values of the determined region-of-interest (ROI). The CST of the whole brain was selected as the ROI. The tractography of the CST was formulated by constraining the seeds at the pons and the bilateral primary motor cortices [29].

### Statistics

Effects of PAS on MEP amplitude, SICI and LICI in the three groups were analyzed by mixed two-way repeated measures analyses of variance (rmANOVAs) with the within-subject factor of TIME (pre- vs. post-PAS) and the between-subject factor of GROUP (PD vs. ET vs. HC). Since sphericity is an important assumption for rmANOVAs, the Greenhouse-Geisser correction was used to correct for any non-sphericity (SPSS 16.0). Conditional on a significant *F* value, *post hoc* between-group comparisons were conducted using Student’s two-tailed *t* test with Bonferroni’s correction for multiple comparisons. *Post hoc* between-time comparisons were conducted by two-tailed paired *t* tests.

For the DTI analyses, the mean FA and the mean MD of the ROI were compared between groups using a non-parametric Kruskal-Wallis test. This method is used for comparing two or more independent samples without an assumption of a normal distribution. Any possible correlations between the PAS effects (i.e. changes in MEP amplitude, SICI and LICI) and the DTI measures (i.e. mean FA and mean MD) in which ROI included the left CST and the bilateral CSTs were examined by linear regression. It is used to approach the relationship between a dependent variable and one or more independent variables. For all tests, the significance level was set to p < 0.05.

## Results

RMT, AMT, the intensity of TMS producing MEPs of on average 1 mV in peak-to-peak amplitude in the resting APB (MEP_1mV_), conditional TMS intensities for SICI and LICI did not show any statistical difference between the three groups (**Table 2**). All subjects showed a normal N20-latency. The N20-latency and the SEP amplitude N20-P25 were also not significantly different between the three groups (**Table 2**).

**Table 2:** Baseline measures of transcranial magnetic stimulation (TMS) and median nerve somatosensory-evoked potential (SEP).

	RMT (%MSO)	AMT (%MSO)	MEP_1mV_* (%MSO)	Conditional TMS intensity for SICI (%MSO)	Conditional TMS intensity for LICI (%MSO)	N20 latency (ms)	N20-P25 amplitude (μV)
**HC**	51.9±7.2	41.8±6.3	74.7±11.3	41.8±6.4	61.9±9.6	19.8±1.3	3.9±3.0
**PD**	48.8±7.6	41.3±6.1	63.3±15.8	40.9±5.9	56.1±8.7	20.7±0.7	4.5±1.8
**ET**	48.8±9.5	41.2±6.0	63.4±14.3	39.2±6.8	54.0±9.9	20.2±1.6	5.1±2.6

* The intensity of TMS producing MEPs of on average 1 mV in peak-to-peak amplitude in the resting abductor pollicis brevis (APB).

Abbreviations: AMT: active motor threshold; ET: essential tremor; HC: health control; LICI: long-interval intracortical inhibition; MSO: maximum stimulator output; PD: Parkinson’s disease; RMT: resting motor threshold; SICI: short-interval intracortical inhibition

**Fig 1:**
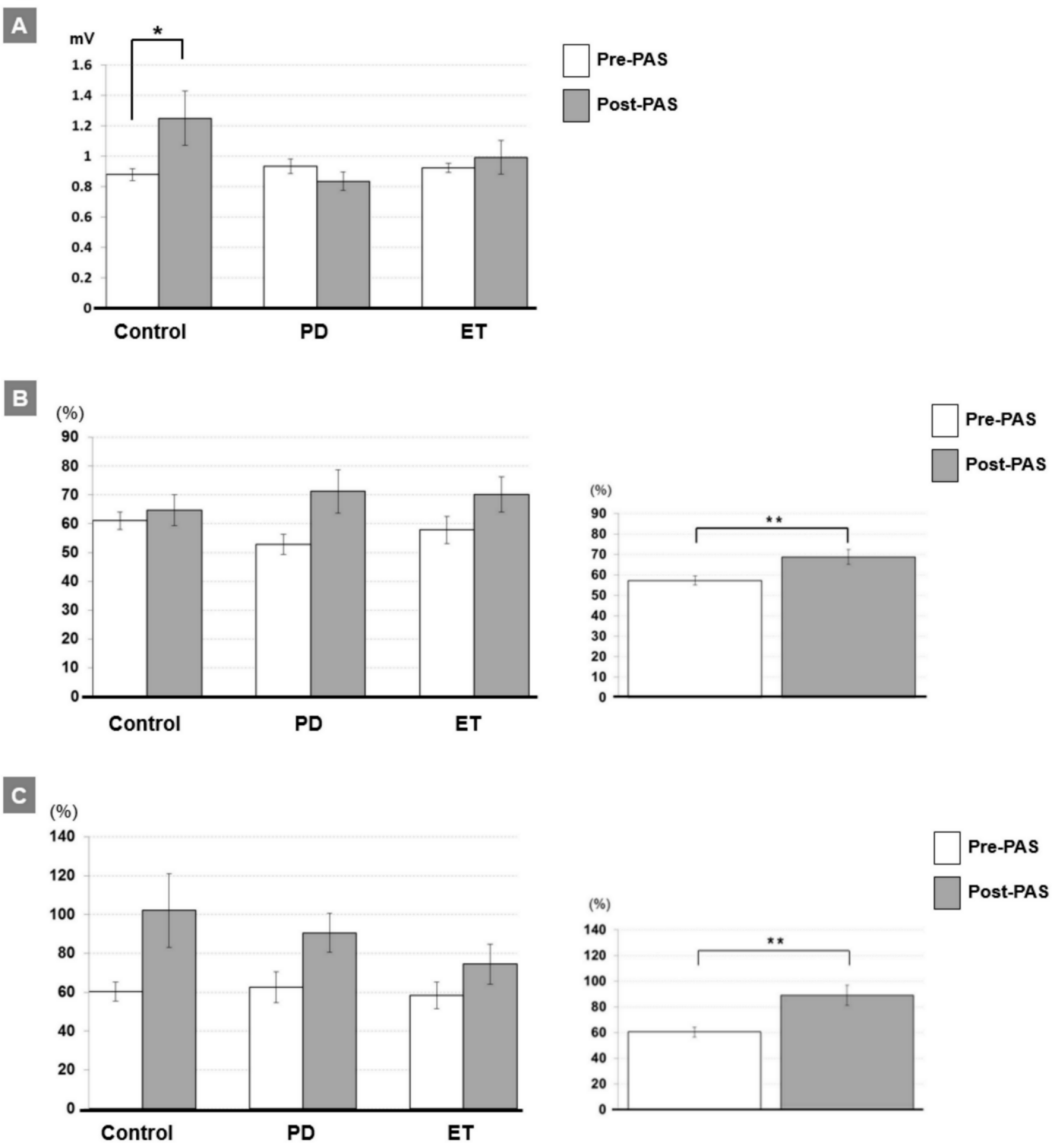
A. MEP amplitudes (in mV) pre-PAS (white columns) vs. post-PAS (gray columns, mean ± S.E.M) recorded from the APB muscle in the three groups. MEP amplitudes significantly increased after PAS in the control group but not in the other two groups (*P < 0.05 by two-tailed paired *t* test). B. Mean SICI (given as percentage of the conditioned MEP/unconditioned MEP) pre-PAS (white column) vs. post-PAS (gray column, mean ± S.E.M) in the three groups (left panel). Overall, a significant reduction of SICI occurred after the PAS intervention irrespective of groups (right panel, **P < 0.01 by two-tailed paired *t* test). C. Mean LICI (given as percentage of the conditioned MEP/unconditioned MEP) pre-PAS (white column) vs. post-PAS (gray column, mean ± S.E.M) in the three groups (left panel). Overall, a significant reduction of LICI occurred after the PAS intervention irrespective of groups (right panel, **P < 0.01 by two-tailed paired *t* test).

The two-way rmANOVA of the MEP demonstrated a significant interaction between TIME and GROUP (F_2,33_ = 4.42, *P* = 0.02; **Table 3**). *Post hoc* testing showed that a significant effect of TIME occurred only in the control group (pre/post: 0.88±0.14/1.25±0.62 mV, *P* = 0.033) but not in the other two groups (both *P* > 0.08) (**Fig 1A**). In addition, the rmANOVAs of the SICI and LICI showed a significant main effect of TIME (both *P* < 0.005 for SICI and LICI; **Table 3**). The effect of TIME in SICI and LICI could be explained by a general reduction of both forms of inhibition after PAS irrespective of groups (pre/post: 57.3±13.3/68.7±21.8% for SICI, *P* < 0.01, **Fig 1B**; 60.4±22.7/89.0±47.7% for LICI, *P* < 0.01, **Fig 1C**). The statistical power reaches 0.95 with the effect size of 0.34 for the two-way rmANOVA.

**Table 3:** RmANOVA of the PAS effect on TMS measures the motor cortical excitability.

		MEP		SICI		LICI	
	*d*.*f*.	*F*	*P*	*F*	*P*	*F*	*P*
*Within-subject factor*							
**Time**^**a**^	1	3.03	0.091	**10.93**	0.0023**	**12.02**	0.0015**
*Between-subject factor*							
**Group**^**b**^	2,33	1.45	0.25	0.054	0.95	0.87	0.43
**Time** X **Group**	2,33	**4.42**	0.02*	1.51	0.24	0.81	0.46

^a^2 levels (pre-PAS and post-PAS)

^b^3 levels (PD, ET and HC)

* *P* < 0.05

** *P* < 0.01

**Fig 2:**
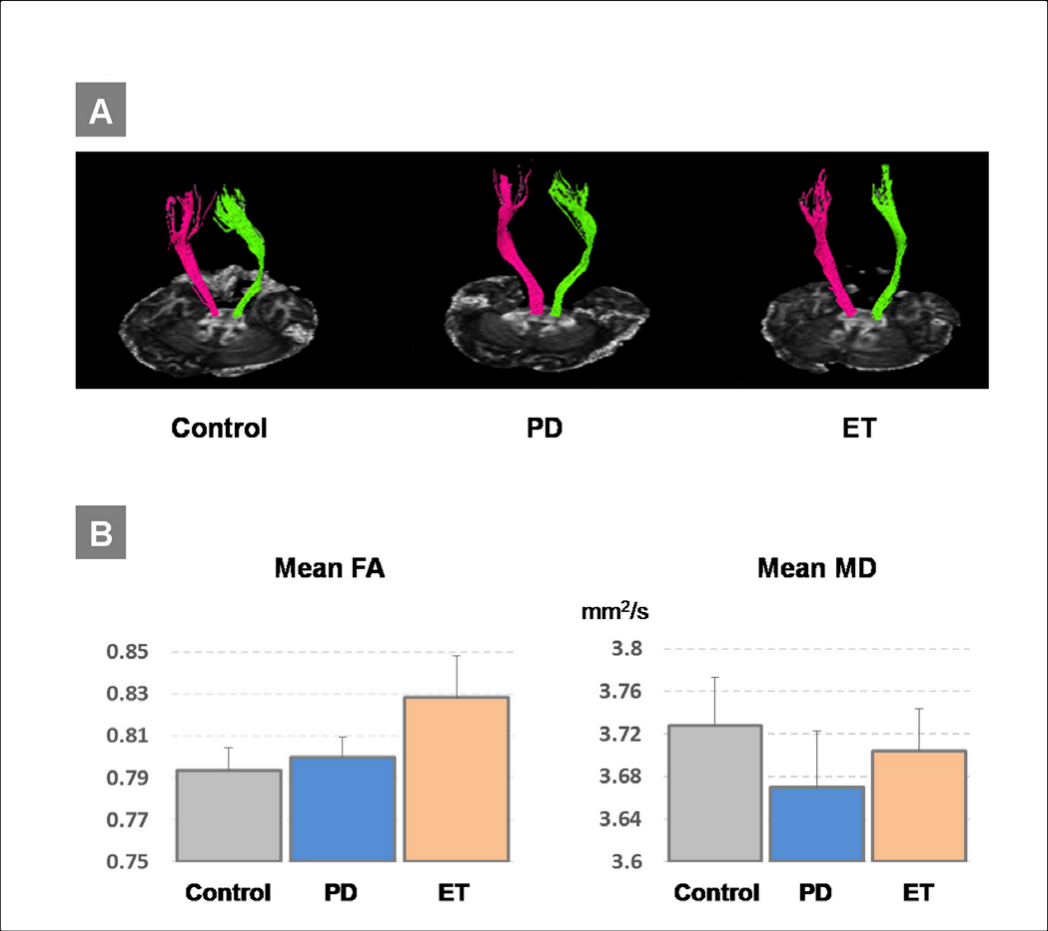
A. Illustration of one representative example of the individual diffusion tensor imaging (DTI) tractography of the corticospinal tract (CST) in each of the three groups. B. Mean fractional anisotropy (FA) and mean diffusivity (MD) of the CST in the three groups (mean ± S.E.M). There was no significant difference for any of these measures between groups (all *P* > 0.3).

No significant differences of the mean FA and the mean MD were found between the groups (all *P* > 0.3 by Kruskal-Wallis test) (**Fig 2**). There was no significant correlation between any of the PAS effects and the DTI measures including the left CST and the bilateral CSTs (all *P* > 0.2). The raw data of the TMS measures, the FA and the MD are appended in the Supporting Information (S1 File).

## Discussion

### Impaired M1 plasticity in PD and ET

The current findings on motor cortical plasticity in PD are consistent with the previous literature showing that PD patients have impaired PAS-induced LTP-like plasticity [30–32]. The PAS-induced LTP-like MEP increase is thought to serve as a model of heterosynaptic plasticity at the systems level [6, 16]. It was found significantly reduced in PD patients off medication [30]. However, the capacity to induce LTP-like plasticity may depend on the employed technique of non-invasive brain stimulation. In contrast to deficient LTP-like plasticity induced by PAS, PD patients showed intact LTP-like plasticity when induced by intermittent theta-burst stimulation [33], a patterned repetitive TMS protocol inducing homosynaptic plasticity [16, 34]. This discrepancy between homosynaptic and heterosynaptic LTP-like plasticity in PD needs further investigation.

There is only a limited number of studies that tested motor cortical plasticity in ET. In a small size case control study, eight ET patients showed a preserved LTP-like plasticity with PAS intervention [31]. A fixed interstimulus interval of 25 ms between the electrical stimulation of the median nerve and TMS of M1 was applied in that study. In the present study the interval was adjusted based on the individual N20 latency (i.e. N20 plus 2 ms). Recent studies have revealed that PAS with a 25 ms-interval and PAS with a 21.5 ms-interval may engage separate mechanisms for LTP-like plasticity induction [33]. Therefore, the findings of the previous study [31] do not allow the general conclusion that M1 plasticity is normal in ET. Another study that applied continuous theta-burst stimulation, a protocol that in healthy subjects leads to a long-term depression (LTD)-like decrease in MEP amplitude [34], demonstrated a lack of this type of LTD-like plasticity in ET patients [35]. The current data support the notion that M1 plasticity is impaired in ET patients with intention tremor. Since PAS with a 25 ms-interval may involve the cerebellar circuits which were supposed to trigger intention tremor in ET [19], one may argue that the PAS with a 25 ms-interval can be more appropriate than the PAS with an interval of N20 plus 2 ms for our patient group. However, the previous report did not support the notion that the PAS with a 25 ms-interval is capable of detecting M1 plasticity change in ET patients with kinetic tremor [31]. A comparison of PAS with the two interval paradigms will be helpful to clarify this issue in the future.

Despite of their distinct subcortical pathogeneses, PD and ET tremor has been supposed to share some common pathophysiology in M1 [2–5]. The current findings also suggest that PD and ET with intention tremor may have a similar impairment of the associative LTP-like corticospinal excitability change in M1. This interpretation is inevitably limited by the current cross-section study design. A longitudinal follow-up and recruitment of distinct subgroup patients with PD and ET are anticipated to make a final conclusion.

Although the MEP_1mv_ was not statistically different between the groups, our data showed a relatively lower mean MEP_1mv_ in the PD and the ET groups compared to the HC group (**Table 2**). For PD patients, the corticospinal excitability represented by input/output (I/O) curves has been found steeper in the more affected M1 than the less affected M1 following the disease progression [36]. This renders a possibility that we could produce MEPs of 1mV with a less TMS intensity in our PD patients with significant motor symptoms than in our HC. Someone may still concern that the relatively low MEP_1mv_ in the patient groups might be caused by subtle muscle contraction during the recording procedures. Accordingly, the current finding on the PAS effect can be influenced by the assumed confounding factor. However, it has been known that the LTP-like effects of PAS are enhanced when the PAS is given during voluntary muscle contraction [37]. Therefore we could only underestimate the impairment of the M1 plasticity in the patient groups.

### PAS-induced SICI and LICI reduction

In general, PAS reduced SICI and LICI (**Table 3**; **Fig 1B and 1C**). This finding seems inconsistent with previous findings that PAS does not change SICI [22, 38, 39]. The SICI response to the excitability-enhancing PAS protocol, however, may depend on the individual baseline SICI [40, 41]. When an evident baseline SICI is induced with a high conditional TMS intensity, the SICI following the PAS intervention can be reduced [40]. In this study we adopted high conditional TMS intensities (see **Table 2**) to determine the baseline SICI as ~50% reduction of the unconditioned MEP amplitude. Such a SICI level would fall into an evident range of SICI [40]. For PD patients during their off state, SICI could be reduced and a maximal SICI was reported around 50–60% reduction of the unconditioned MEP amplitude [42–46]. Although we did not measure the SICI recruitment curve with several steps of the conditional TMS intensities, it is likely that the baseline SICI recorded in this study already reaches a maximal level, particularly for the PD patients.

LICI was reported to be increased by excitatory PAS [23, 30, 40]. It was found significantly decreased only by a “low intensity” TMS setting, e.g. evoking a test MEP of 0.5 mV [47]. Another possible explanation for the discordant findings between the current data and the previous reports could be the age of the tested subjects, as the mean age in this study was considerably higher than the previous reports, and age is known to have a significant influence on cortical plasticity [48].

Patients with ET have been reported with normal SICI and LICI [49, 50]. However, the baseline SICI and LICI can be already abnormal in patients with PD [43–46, 51]. The current findings cannot rule out the possibility that the PAS intervention has different influences on corticospinal excitability and intracortical inhibitions between PD and ET. Further studies concerning the PAS effect on different cortical neurons are anticipated to clarify these influences in PD and ET.

### Diffusion tensor image of the CST

The DTI findings in this study do not suggest the CST as a main pathological target for either PD or ET. Distinct from other Parkinsonian syndromes, such as multiple system atrophy and progressive supranuclear palsy, PD patients did not show significant CST white matter abnormality [13]. ET patients revealed abnormalities in the anterior limb of the internal capsule and in the cerebellar peduncles but not in the CST [12]. The current DTI findings in PD and ET patients are in agreement with those previous reports. The lack of any correlation between the PAS effects and the DTI findings further suggests that the microstructure of the CST does not play a significant role in the generation of the STDP-like plasticity. Future work focusing on the microstructure within the sensorimotor cortex (e.g. high resolution U fibers that connect somatosensory with motor cortex) might be more revealing in establishing a relation with PAS-induced plasticity, as it is thought that this form of plasticity is being mediated through this connection [52].

## Conclusions

This study compared associative motor cortical plasticity in patients with PD, patients with ET with intention tremor and healthy controls, and the relationship of this plasticity with CST microstructure. Findings suggest that both PD and ET with intention tremor have impairment of the associative LTP-like corticospinal excitability change in M1. The microstructure of the CST was found to be intact in PD and ET and, thus, the CST is probably not relevant to the observed deficient plasticity in these patients.

## Supporting Information

S1 File Raw data of the TMS and the DTI measures. (XLS)

S1 Text Abstract for the 1st International Brain Stimulation Conference in Singapore (2015). (PDF)

## Abbreviations

AMT: active motor threshold
APB: abductor pollicis brevis
CST: corticospinal tract
DTI: diffusion tensor imaging
ET: essential tremor
FA: fractional anisotropy
HC: healthy control
ISI: interstimulus interval
LICI: long-interval intracortical inhibition
LTD: long-term depression
LTP: long-term potentiation
M1: primary motor cortex
MD: mean diffusivity
MEP: motor evoked potential
PAS: paired associative stimulation
PD: Parkinson’s disease
RMT: resting motor threshold
ROI: region-of-interest
SEP: somatosensory-evoked potential
SICI: short-interval intracortical inhibition
STDP: spike timing-dependent plasticity
TMS: transcranial magnetic stimulation
