# Supplementary material for: Investigation of Motor Cortical Plasticity and Corticospinal Tract Diffusion Tensor Imaging in Patients with Parkinsons Disease and Essential Tremor
Source: PLoS One. 2016 Sep 7;11(9):e0162265. doi: 10.1371/journal.pone.0162265 (PMC5014415; doi:10.1371/journal.pone.0162265)
Supplement: S1 Text — (PDF) [file pone.0162265.s002.pdf]

**Background:** Non-invasive brain stimulation (NIBS) protocols can interfere with or even facilitate motor learning. However, it is still unclear whether the response to NIBS protocols, such as theta-burst stimulation (TBS), can predict its ability to interfere with or enhance learning of a motor task. We aim to investigate this question using TBS and a ballistic finger movement task.
**Methods:** Eight right handed healthy human volunteers participated in the study. Experiment 1: We investigated the effects of continuous TBS (cTBS) and intermittent TBS (iTBS) on cortical excitability. Thirty motor evoked potentials (MEP) were measured before and after intervention (cTBS or iTBS) for 30 min. Experiment 2: In separate sessions, we tested the effects of cTBS or iTBS on a ballistic finger movement task. Subjects were required to make rapid index finger abduction movements of the right hand paced at a rate of 0.5 Hz. One block consists of 75 movements for 2.5 min and 4 blocks were tested in total. Between blocks 2 and 3, either cTBS or iTBS was applied over left M1. We quantified the learning effect by measuring the increase the mean peak acceleration (ACC) for each set of 30 consecutive movements.
**Results:** In experiment 1, there was a non-significant trend for MEPs to get smaller after cTBS and larger after iTBS. In experiment 2, cTBS or iTBS did not change ACC immediately after application of TBS. However, ACC increased significantly more in the final block of trials after cTBS than it did after iTBS. The normalized ACC in the last block was negatively correlated with excitability changes induced by cTBS in experiment 1 (Spearman's $\rho = 0.881$, $p = 0.002$).
**Conclusions:** This preliminary result suggest that cTBS did not affect early consolidation in M1, but facilitated further performance gains in model-free learning. The effect on performance was correlated with the effect of cTBS on MEPs.

## 115

### Motor cortical plasticity and corticospinal tract diffusion tensor image in patients with Parkinson's disease and essential tremor

*M.K. Lu* [a,b,*], *C.M. Chen* [c], *J.R. Duann* [b,d], *U. Ziemann* [e], *C.H. Tsai* [a,b]
[a] *Neuroscience Laboratory, Department of Neurology, China Medical University Hospital*
[b] *School of Medicine, Medical College, China Medical University*
[c] *Department of Radiology, China Medical University Hospital*
[d] *Biomedical Engineering Research Center, China Medical University, Taichung, Taiwan*
[e] *Department of Neurology & Stroke, Hertie Institute for Clinical Brain Research, Eberhard-Karls-University, Tübingen, Germany*
*E-mail: d4297@mail.cmuh.org.tw.

**Introduction:** Motor circuits perturbation underlies symptoms in Parkinson's disease (PD) and essential tremor (ET). Paired associative stimulation (PAS)-induced long-term potentiation (LTP)-like plasticity and diffusion tensor image (DTI) of the corticospinal tract (CST) may serve as complementary tools for investigating motor circuits abnormalities in PD and ET.
**Methods:** Ten PD patients (modified Hoehn and Yahr stage 2-2.5, age 67.5±9.0 years), ten ET patients with intention tremor (age 63.4±8.7 years) and ten healthy control (HC) subjects (age 68.7±7.1 years) were studied. PAS consisted of 225 pairs of electrical stimulation of the right median nerve followed by a single transcranial magnetic stimulation (TMS) pulse over the left primary motor cortex (M1). The interval between electrical stimulation and TMS was 2 ms longer than the individual N20 latency, which is supposed to induce a LTP-like plasticity in M1. Cortical excitability measured by motor-evoked potential (MEP), short-interval and long-interval intracortical inhibition (SICI and LICI) was compared among the three groups before and after PAS intervention. The DTI measurements on fractional anisotropy (FA), mean diffusivity (MD) and fiber number of the CST were done for all participants. The PAS effect and the DTI data were compared between groups.
**Results:** MEP was significantly facilitated by the PAS intervention in the HC group but not in the PD and ET groups. SICI and LICI showed a non-specific reduction after the PAS intervention across groups. No significant differences of the mean FA, MD and fiber number of the CST were found between groups.
**Discussion:** Findings suggest that PD and ET with intention tremor may share a similar defect on the motor cortical plasticity induced by PAS. The white matter of the CST is probably not relevant to the defect in the PD patients and the ET patients with intention tremor.

## 116

### Transcranial direct current stimulation over the prefrontal cortex alters reinforcement of trial-and-error behavioural learning

*Takayuki Kawaguchi* [a], *Makoto Suzuki* [a], *Makoto Watanabe* [b], *Shinobu Shimizu* [b], *Kazuhiko Shibata* [a], *Aki Watanabe* [a], *Kayoko Takahashi* [a], *Michinari Fukuda* [a]
[a] *Graduate School of Medical Sciences, Kitasato University*
[b] *School of Allied Health Sciences, Kitasato University*

**Introduction:** The left prefrontal cortex is known to play a key role in trial-and-error learning process that selects between competing reward and cost. This study investigated a shift of the performance of trial-and-error behavioural tasks by transcranial direct current stimulation (tDCS) to the left prefrontal cortex.
**Methods:** Before the behavioural tasks, participants were stimulated with tDCS (2 mA) for 20 min. For stimulation the prefrontal cortex, the anode or cathode electrode was placed over left F3 (international EEG 10/20 system). In sham experiments, tDCS was turned off after 30 s. Each behavioural trial began with one of three colored circles presented as a cue. Two seconds after the presentation of the cue, the reward/cost stimulus was randomly presented for a duration of 2 s as a feedback to the subject. Each color showed 10%, 50% or 90% reward probability. The subject was required to decide as quickly as possible whether to perform wrist flexion in response to the color of the circle, and if the picture of a coin appeared after wrist flexion, the subject received the actual coin after the experiment. However, if a mauve circle appeared after the wrist flexion, the coin was deducted from the total reward per occurrence.
**Results:** Cumulative numbers of behaviours for 50% and 90% reward probabilities were not significantly different among anode, cathode and sham tDCS. However, cumulative numbers of behaviours for 10% reward probabilities were more in anode tDCS than in cathode and sham tDCS.
**Discussion:** Anode tDCS for left-prefrontal cortex induced a decrease in sensitivity for the cost and a shift to the performance of commission errors. These results imply that trial-and-error behavioral learning including the reward-related circuit might be altered by tDCS.
